# Prevalence, Risk Factors, and Antibiogram of Nontyphoidal *Salmonella* from Beef in Ambo and Holeta Towns, Oromia Region, Ethiopia

**DOI:** 10.1155/2021/6626373

**Published:** 2021-03-23

**Authors:** Endrias Zewdu Gebremedhin, Gezahegn Tafesse Soboka, Bizunesh Mideksa Borana, Lencho Megersa Marami, Edilu Jorga Sarba, Nega Desalegn Tadese, Hirut Abebe Ambecha

**Affiliations:** ^1^Ambo University, College of Agriculture and Veterinary Sciences, Department of Veterinary Sciences, P.O. Box 19, Ambo, Ethiopia; ^2^West Shewa Zone, Ambo District, Ambo, Ethiopia; ^3^Ambo University, College of Agriculture and Veterinary Sciences, Department of Veterinary Laboratory Technology, P.O. Box 19, Ambo, Ethiopia

## Abstract

**Background:**

*Salmonella* has been recognized as a major cause of food-borne illness associated with the consumption of food of animal origin. The present cross-sectional study was conducted from December 2017 to May 2018 in Ambo and Holeta towns to assess the prevalence, risk factors, and antimicrobial susceptibility patterns of nontyphoidal *Salmonella* isolates from raw beef samples from abattoirs, butchers, and restaurants in Ambo and Holeta towns, Oromia region, Ethiopia.

**Methods:**

A total of 354 beef samples were collected from abattoirs, butchers, and restaurants. *Salmonella* isolation and identification were carried out using standard bacteriological methods recommended by the International Organization for Standardization. Antimicrobial susceptibility testing was performed using the disk diffusion method. Besides, a structured questionnaire was used to collect sociodemographic data and potential risk factors for contamination of meat. Chi-square tests and logistic regression were used for data analyses.

**Results:**

Of the total 354 meat samples examined, 20 (5.7%, 95% confidence interval (CI): 3.5–8.6) were positive for *Salmonella*. Two serotypes belonging to *S. typhimurium* (11 isolates) and I:4,5,12: i:- (9 isolates) were identified. The *Salmonella* detection rate in abattoirs, butchers, and restaurants was 4.2% (5/118), 8.5% (10/118), and 4.2% (5/118), respectively. The antimicrobial susceptibility test showed that 40%, 30%, and 20% of the *Salmonella* isolates were resistant to azithromycin, amoxicillin, and ceftriaxone, respectively. The odds of *Salmonella* isolation when meat handlers are illiterate were 7.8 times higher than those when they are educated to the level of secondary and above (*P*=0.032). Similarly, the likelihood of *Salmonella* isolation was 6.3 and 7.6 times higher among workers of butcher and restaurants, respectively, who had no training (*P*=0.003) and no knowledge (*P*=0.010) on food safety and hygiene.

**Conclusions:**

The study showed widespread multidrug-resistant *Salmonella* isolates in the study areas. Therefore, raw meat consumption and indiscriminate use of antimicrobial drugs should be discouraged. Provision of food safety education for meat handlers and further surveillance of antimicrobial-resistant isolates are suggested.

## 1. Introduction

Food-borne bacterial diseases are severe challenges to human and animal health globally. Contaminated food of animal origins such as poultry, pork, beef, and dairy products is often linked to cases of human salmonellosis [1]. Among food-borne pathogens, *Salmonella* is one of the most important bacteria causing gastroenteritis in humans and animals. It is the leading cause of food-borne diarrhoeal disease and an important public health problem worldwide particularly in developing countries [2]. The epidemiology of these diseases has been modified tremendously due to globalization of trade, urbanization, change in consumers' behaviors, increased population, demographic changes, changes in industrial structure, and the capacity of the pathogens to acclimatize to new situations [3, 4].

*Salmonella*, which belongs to the family Enterobacteriaceae, is a Gram-negative, facultatively anaerobic, and non-spore-forming motile rod-shaped bacterium. The bacteria are mostly nonlactose fermenters, oxidase negative, catalase-positive, produce acid and gas from glucose, and utilize citrate as a sole carbon source. *Salmonella* are classified and identified in 7 subspecies [5]. Currently, the genus *Salmonella* comprises only two species: S. *enterica* and S. *bongori*. The former is further divided into six subspecies, viz., *Salmonella enterica* subsp. e*nterica, Salmonella enterica* subsp. *Arizonae*, *Salmonella enterica* subsp. *diarizonae, Salmonella enterica* subsp. *houtenae, Salmonella enterica* subsp. *indica*, and *Salmonella enterica* subsp. *salamae* [5, 6]. Serotypes belonging to *Salmonella enterica* subsp. *enterica* are responsible for about 99% of *Salmonella* infections in humans and warm-blooded animals, while the other five subspecies and *Salmonella bongori* are predominantly found in cold-blooded animals and the environment [7]. There are over 2,610 *Salmonella* serovars [8]. Depending on the host, S. *enterica* serovar *typhimurium* (*S. typhimurium*) can cause diseases ranging from gastroenteritis to life-threatening systemic infection [6].

*Salmonella* is estimated to cause 93.8 million human gastroenteritis infections and 155, 000 deaths each year worldwide [9]. Although most of these infections cause mild gastroenteritis, life-threatening dispersed infections are common among children, the elderly, and immunocompromised patients [10]. Various animals, including poultry, pigs, cattle, and reptiles, are reservoirs of *Salmonella* species. A majority of human *Salmonella* infections are developed after the ingestion of undercooked food of animal origin or contaminated water and vegetables [9].

*Salmonella enterica* serovars *typhimurium* and *enteritidis* have been the major causes of human salmonellosis in developed nations. However, in speciﬁc geographical regions, cases of human salmonellosis due to other serovars such as S. Stanley and S. Weltevreden in South-East Asia are common [11]. *Salmonella* can also spread through the stool of human beings which serves as an important reservoir of *Salmonella* serovar. Human illness, as well as the spread of *Salmonella*, could take place when food and water are contaminated with the stool or directly via the feco-oral route [12].

At the butchers' house, meat contamination could occur due to different possible reasons such as storing food in unclean utensils, holding food at a temperature that would allow microbial growth, utilization of poor-quality water, using packaging materials that are not of food-grade quality, a retailing site that had no facilities for waste disposal, and utilization of unclean utensils. Besides, the lack of awareness in basic personal cleanliness and safe food handling of butchers increases the contamination of beef by microorganisms [13]. Meat is a highly perishable food. The rich source of nutrients of fermented meat offers microbes an appropriate milieu to propagate throughout the preparatory step and storage. An upsurge in the demand for beef devoid of the required setup for appropriate healthy handling might lead to the transmission of disease-causing microorganisms from the animals to the consumer [14].

Contamination of meat by *Salmonella* could occur at abattoir from the excretion of asymptomatic animals, unclean abattoir tools, floor, and workers. The pathogens enter the meat at any stage during slaughtering. Cross-contamination of carcasses and meat products could continue during successive handling, processing, preparation, and delivery [15].

The significance of salmonellosis in the public health sector is a mounting concern day by day worldwide [16]. Salmonellosis frequently follows the ingestion of food of animal origin such as raw meat. Retailing raw meat at butcher shops is extensively accomplished in different towns of Ethiopia including the study areas. Butcher shops are the chief provider of meat of different animals to buyers. Failure to carefully clean work surfaces used to prepare raw meat and other foods in the restaurants can also be a source of *Salmonella*. Moreover, in Ethiopia, minced beef is usually used for the preparation of a popular traditional Ethiopian dish known as “Kitfo” (minced raw beef mixed with a chili powder-based spice blend and a clarified butter infused with herbs and spices), and most of the time, it is consumed raw or medium-cooked. The renowned custom of raw meat consumption, the presence of *Salmonella* in minced beef, and the inadequate hygienic standards in food handling indicate the likely occurrence of public health hazards due to *Salmonella* [17, 18]. This study aimed to estimate the prevalence, antimicrobial susceptibility patterns, and risk factors of nontyphoidal *Salmonella* in raw beef along the meat chain in Ambo and Holeta towns, Oromia, Ethiopia.

## 2. Materials and Methods

### 2.1. The Study Area

This study was conducted in Ambo and Holeta towns that are located 115 and 29 kms west of Addis Ababa, respectively. Ambo town is found between 8°56′30“ N–8°59′30” N latitude and 37°47′30“ E–37°55'15” E longitude. Holeta is located at approximately 09° 03′–19.43“ N latitude and 38° 30′–25.43”E longitude. There are two municipality abattoirs (one in each town) and 92 butcher shops in Ambo (*n* = 72) and Holeta (*n* = 20) towns (Ambo town trade and marketing office, 2017). Stunning, evisceration, and managing of visceral organs were performed in the same room in Ambo as well as Holeta municipality abattoirs.

### 2.2. Study Design and Population

A cross-sectional study was conducted from December 2017 to May 2018. The study populations were cattle slaughtered for human consumption in Ambo and Holeta municipality abattoirs and sold in butcher shops and restaurants operating in the two towns during the study period.

### 2.3. Sample Size and Sampling Technique

The sample size required was calculated using 17.3% expected prevalence [19] with a 5% level of precision and 95% confidence interval using the formula described as follows [20]:

(1) $$\begin{array}{c} N=\frac{1.96^{2}\text{Pexp}\left(1-\text{Pexp}\right)}{d^{2}}, \end{array}$$

where, *N* = sample size, P exp = expected prevalence, and *d* = absolute precision. Accordingly, the minimum sample size calculated was 220. Nevertheless, 354 samples were taken deliberately to reduce sample losses during processing and maximize the precision of the study. Of the total meat samples collected, abattoirs, butchers, and restaurants each accounted for 118 samples. The total sample size (*n* = 354) was proportionally distributed to the two towns based on the number of butcher shops that are found in the two towns, i.e., 267 in Ambo and 87 in Holeta towns (1 : 3 ratios). Systematic random sampling was used to sample cattle in the abattoirs.

### 2.4. Study Methodology

#### 2.4.1. Sample Collection and Transportation

Fresh raw meat samples of cattle origin were collected from different portions of the carcass (neck/brisket, fore rib, flank, and rump-followed by pooling) [21] from the abattoir, from displayed meat in the butcher shops, and from restaurants. Samples were kept in separate sterile plastic bags (Seward, England) to prevent spilling and cross-contamination, labeled, and immediately transported to the Ambo University Zoonoses and Food Safety Laboratory in a cooler icebox with ice packs and processed within 4 hrs.

#### 2.4.2. Isolation and Identification of Nontyphoidal *Salmonella* (NTS)

Detection of NTS in the meat samples was performed according to the standard culture method (ISO-6579; 2002). A 25 g meat sample was pre-enriched into 225 ml of buffered peptone water (HiMedia, India) and incubated at 37°C for 24 hrs. Subsequently, 0.1 ml of the pre-enrichment culture was added to 10 ml of Rappaport–Vassiliadis broth (HiMedia, India) and 1 ml to 10 ml of Mueller Kauffman tetrathionate broth (Microgen, India) and incubated for 24 hrs at 41.5°C and 37°C, respectively. The culture was then streaked on two selective agars, xylose lysine desoxycholate (XLD) (Accumix, Belgium) and brilliant green agar (BGA) (Accumix, Belgium), and incubated at 37°C for 24 hrs. The formation of red colonies with black centers on XLD and pink colonies on BGA plates was inspected and considered as presumptive *Salmonella* colonies. For confirmation, up to five presumptive *Salmonella* colonies from both XLD and BGA agars were selected and streaked onto the surface of predried nutrient agar (HiMedia, India) plates and incubated at 37°C for 24 ± 3 hrs. Colonies from nutrient agar were inoculated into the following biochemical tubes for identification: triple sugar iron (TSI) agar (Accumix, Belgium), lysine iron agar (Pronadisa, Spain), Simmon's citrate agar (HiMedia, India), urea agar (HiMedia, India), and indole reaction MIO (motility indole ornithine) medium (Pronadisa, Madrid, Spain) and incubated for 24 or 48 hrs at 37°C.

### 2.5. Antimicrobial Susceptibility Test

Nontyphoidal *Salmonella* isolates were subjected to *in vitro* antimicrobial susceptibility tests against commonly used antimicrobial drugs using the disk diffusion method following guidelines established by the Clinical and Laboratory Standards Institute [22]. In brief, each isolated bacterial colony from pure fresh culture was transferred into a test tube of 5 ml tryptone soya broth (HiMedia, India) and incubated at 37°C for 6 hrs. The turbidity of the culture broth was adjusted using a sterile saline solution or by added more isolated colonies to obtain turbidity usually comparable with that of 0.5 McFarland standards (approximately 3 × 10^8^ CFU per ml). The diluted bacterial suspension was transferred to the Mueller–Hinton agar (HiMedia, India) plate using a sterile cotton swab, and the plate was seeded uniformly by rubbing the swab against the entire agar surface followed by 24 hrs incubation. After the inoculums, dried, antimicrobial impregnated disks were applied to the surface of the inoculated plates using sterile forceps. The plate was incubated aerobically at 37°C for 24 hrs. Finally, the zone of inhibition was measured using a caliper by including the disk diameter. The susceptible, intermediate, and resistant categories were assigned based on the critical points recommended by the CLSI [22] and according to the manufacturer's leaflet attached to the disks. Standard strains of *E. coli* ATCC 29522 and ATCC 35218, which are susceptible to all the drugs kindly provided by the Ethiopian Public Health Institute, were used as quality control organisms for the antimicrobial susceptibility test.

### 2.6. *Salmonella* Serotyping

Serotyping of *Salmonella* isolates was done at the Public Health Agency of Canada, World Organization for Animal Health (OIÉ) Reference Laboratory for Salmonellosis, Guelph, Ontario, Canada (report number: UAA160121118R September 12, 2018). In brief, the somatic (O) antigens were determined by slide agglutination tests, and flagella antigens were determined using a microplate agglutination technique [23]. The antigenic formulae of Grimont and Weill [24] were used to identify and assign the serotypes of the isolates.

### 2.7. Questionnaire Survey and Observations

A structured questionnaire was administered for 269 people working in abattoirs (33), butchers (118), and restaurants (118). The questionnaire survey was administered to assess the potential risk factors for contamination of meat and the knowledge and practice on food hygiene and food safety of workers in abattoirs, butchers, and restaurants. In brief, the questions administered included age in years (<20, 21–29, 30–39, and ≥39), sex (male and female), religion (Orthodox, Protestant, and others), marital status (single, married, and divorced), residential place (urban and rural), level of education (primary, secondary, and tertiary schools), source of meat (abattoirs, butchers, and restaurants), work experience in years, time spent on work per day, information about food hygiene and safety (yes/no), training on food safety and hygiene (yes/no), knows food safety and hygiene (yes/no), and practice of food safety and hygiene (yes/no). Moreover, using observational checklists, sanitation of butchers and restaurants, hygiene of slicing materials, cutting boards, and food handlers were rated as poor, fair, and good.

### 2.8. Variables

Dependent variables: prevalence and antimicrobial susceptibility pattern of *Salmonella*.

Independent variables: sociodemographic characteristics (sex, age, religion, marital status, educational status, and residence) of food handlers, knowledge about food safety and hygiene (food-borne disease, food contamination, and microorganisms), hygiene and sanitation-related problems of the toilet, water source, waste management, handling practice, and general cleanliness of rooms, dining tables, hand washing basins, and utensils cleaning techniques.

### 2.9. Data Analysis

The questionnaire and laboratory data were entered into Microsoft Excel Spreadsheet. Data were entered and analyzed using the statistical software STATA version 11.0. Descriptive statistics were used to describe the frequency of *Salmonella* from different sampling points, antimicrobial susceptibility patterns, and hygienic conditions. The sample level prevalence was calculated as the number of samples positive for *Salmonella* divided by the total number of samples examined multiplied by 100. Initially, the association between each exposure variable and the presence of *Salmonella* was assessed using the Chi-square test. Univariable and multivariable logistic regression analyses were done, and crude and adjusted odds ratios with 95% confidence interval (CI) were calculated for statistical significance tests. Noncollinear variables with *P* value <0.25 in a univariable logistic regression analysis were considered for multivariable analysis to look for a relative effect on the outcome variable by controlling other possible confounding factors. The binomial exact method was used to calculate the 95% confidence interval (CI) of the prevalence estimates. *P* value <0.05 was considered statistically significant.

## 3. Results

### 3.1. Prevalence of *Salmonella*

Out of 354 samples tested, the prevalence of nontyphoidal *Salmonella* was found to be 5.7% (95% CI = 3.5–8.6%). The prevalence of *Salmonella* contamination in Ambo and Holeta towns was 7.5% (95% CI = 4.6–11.3%) and 0%, respectively (Table 1).

### 3.2. *Salmonella* Serotype Distribution

Of the 20 isolates subjected to the serotyping, all isolates were confirmed to be *Salmonella*. Overall, 2 different *Salmonella* serotypes were recovered, the predominant serotype being *S. typhimurium* followed by I:4,5,12:i:- (Table 2).

### 3.3. Antimicrobial Susceptibility of *Salmonella*

*Salmonella* isolates were subjected to an antimicrobial susceptibility test using 15 selected antimicrobial drugs (Table 3). The isolated strains were 100%, 95%, 95%, and 80% susceptible to cotrimoxazole, chloramphenicol, norfloxacin, and nitrofurantoin, respectively. In other cases, 40%, 30 %, and 25% of the *Salmonella* isolates were resistant to azithromycin, amoxicillin, and nalidixic acid, respectively (Table 3).

*Salmonella* isolates showed sixteen different antimicrobial resistance patterns (Table 4). Out of 20 *Salmonella* isolates, 6 (30%) developed resistance to three or more classes of antimicrobial drugs, i.e., multidrug resistance (MDR). One isolate (5%) had developed resistance to five classes of antimicrobials. Nine isolates (45%) of *Salmonella* isolates were resistant to at least two classes of antimicrobial drugs.

### 3.4. Sociodemographic Characteristics of the Workers

The sociodemographic information of abattoir, meat retailer, and restaurant workers interviewed in Ambo and Holeta towns showed that most of the respondents from abattoirs (66.7%), retailer shops (51.7%), and restaurants (50.9%) were between the ages of 21 and 29 years. A majority (94.9%) of the workers in the restaurants were females. On the other hand, the majority of the workers in abattoirs (51.5%), butcher shops (67.8%), and restaurants (56.8%) had primary school level education (Table 5).

### 3.5. Knowledge and Practice on Food Hygiene and Food Safety

Generally, 93.9%, 72.0%, and 83.1% of the respondents working in abattoirs, butchers, and restaurants have information about food hygiene and safety, respectively. About, 66.7%, 44.9%, and 66.1% of the respondents working in abattoirs, butchers, and restaurants, respectively, know about food hygiene and safety, while 45.5%, 42.4%, and 68.6% of them working in the respective places had a good practice on food safety and hygiene (Table 6). During our observation, the gates of the Ambo town abattoir were always open without any restriction to personal movement to go inside and out of the abattoir, and the hygiene of the surroundings and its interior of the abattoir were poor. Moreover, 51.7% of the butcher shop workers and 38.1% of the restaurant workers did not cover their hair while performing their duties at their working place. It has been also observed that the retailer operators retailing meat wipe their hands, cutting board, and weighing balance surfaces with dirty reusable clothes. The same piece of cloth was used throughout the day for drying hands, knives, and chopping boards. Most (56.8%) of the butcher workers did not wash their hands after handling the money. Besides, observational findings showed the absence of washing basins and first aid kits in almost all butchers and restaurants.

### 3.6. Potential Risk Factors for Meat Contamination

Univariable logistic regression analysis revealed that religion (*P*=0.018), education (*P*=0.028), training on food safety and hygiene (*P*=0.001), and time spent on the work per day (*P*=0.008) were significantly associated with the isolation rate of *Salmonella*. At the same time, knowledge of food safety and hygiene (*P*=0.004), sanitation of butcher/restaurants (*P*=0.009), hygiene of slicing materials (*P*=0.003), and hygiene of food handlers (*P*=0.002) were also significantly associated with the isolation rate of *Salmonella*. In this study, the univariable analysis showed that the isolation of *Salmonella* was 7 times higher in food establishments where food handlers are illiterate than the others (OR = 6.8, 95% CI = 1.2–37.7, *P*=0.028). In establishments where the food handlers had no previous training on food safety and hygiene, isolation of *Salmonella* was 6 times higher than those who had the training (OR = 6.03, 95% CI = 2.0–17.9, *P*=0.001). The duration of time spent in the handling of meat per day had also a role in the isolation of *Salmonella*. The likelihood of isolation was nearly 8 times higher in an establishment where the food handlers worked more than 13 hrs per day as compared to those who spent 8–12 hrs per day (OR = 7.7; 95% CI = 1.7–35.0, *P*=0.008). The knowledge of the food handlers on food safety and hygiene also had an impact on the isolation rate of *Salmonella*. In this study, the probability of isolation of *Salmonella* was 9 times higher in those establishments where food handlers had no knowledge of food safety and hygiene than those who knew (OR = 9.11, 95% CI: 2.0–41.4, *P*=0.004). The odds of isolating *Salmonella* was also nearly 6 times higher in butchers and restaurants where the sanitation was poor (OR = 5.8, 95% CI = 1.6–21.8, *P*=0.009). The poor hygiene of the slicing material and food handlers were 8 and 10 times more likely to yield isolation of *Salmonella* when compared with fair and good hygiene (OR = 7.9, 95% CI = 2.0–31.8, *P*=0.003 and OR = 10, 95% CI = 2.5–46.1, *P*=0.002), respectively (Table 7).

All the independent variables investigated were noncollinear with each other except sex of the respondent versus the meat source (*r* = 0.9), the practice of food safety and hygiene of the meat handlers versus their knowledge on food safety and hygiene (*r* = 0.9), and hygiene of the food handlers versus the hygiene of cutting board (*r* = 0.8). Meat source, knowledge of the workers, and hygiene of the food handlers were selected for entry into the multivariable model.

In the univariable logistic regression analysis, source, age, religion, education, food safety training, time spent on the work per day, food safety and hygiene knowledge, sanitation of the butcher/restaurants, hygiene of the slicing materials, and hygiene of the food handlers were associated with the isolation rate of *Salmonella* at a *P* value of ≤0.25. On the other hand, the multivariable logistic regression analysis revealed that the age of the meat handlers, the educational background of the food handlers, the food handlers training on food safety and hygiene, and food safety and hygiene knowledge were significantly and independently associated with the isolation of *Salmonella*, *P* < 0.05. The odds of isolating *Salmonella* was 8 times (*P*=0.049) higher in bluchers and restaurants where the workers did not attend formal education compared to those who attended secondary school and above. Likewise, isolation of *Salmonella* was 10.5 times higher in butchers and restaurants where the food handlers had no food safety and hygiene knowledge (*P*=0.006). The likelihood of isolating *Salmonella* was 5.7 times higher (*P*=0.008) where the food handlers had no previous training on food safety and hygiene than those who had the training (Table 8).

Model selection to identify the best-fitting model showed the level of education of the workers, training of the workers on food safety, and knowledge on food safety and sanitation to be the independent predictors of *Salmonella* isolation (Table 9). The data fitted well the model (Hosmer–Lemeshow Chi-square = 5.85, *P*=0.3210, area under curve (ROC) = 0.8730).

## 4. Discussion

The present study was conducted to estimate the prevalence of *Salmonella* and assess associated risk factors for contamination and antimicrobial susceptibility profile of isolates from meat samples collected from abattoirs, butchers, and restaurants in Ambo and Holeta towns.

Reports from other parts of the world showed varying prevalence, the serotype of *Salmonella*, and antimicrobial resistance levels from raw beef. In developed countries, a prevalence of 0.6% to 4.2% *Salmonella* serotypes in raw beef was reported in Germany [25], Canada [26], and the USA [27, 28]. Relatively higher contamination rates of *Salmonella* in retail beef samples were reported from Malaysia (9.6%) [29], China (17%) [30], and Egypt (8.8%) [31]. In most of these reports, they also found moderate to high MDR *Salmonella* isolates. The difference in the occurrence of *Salmonella* in beef among countries could be partly due to the variation in the hygienic meat handling and processing practice, the types of samples analyzed (whole carcass or steaks or frozen or fresh), and the sensitivity diagnostic test applied.

The overall prevalence of *Salmonella* in the current study towns (5.7%) was higher than that reported from Addis Ababa abattoir enterprise [32], Hawassa [33], Addis Ababa University student's lunchroom [34], and Gondar University [35], which reported a prevalence of 4.6%, 4%, 3.5%, and 3.1%, respectively. A much lower than the present study was also reported from Jimma (1.2%) [35] and central Ethiopia (2.3%) [36].

However, the prevalence of *Salmonella* in this study was lower than the prevalence reported in other studies in Ethiopia such as 10.8% in Jimma [37], 12.9% in Addis Ababa [38], 12.5% in Wolaita Sodo [39], 14% in central Ethiopia [40], 15% in Bahir Dar [41], 17.3% in Gondar town [19], and 17.5% in Arba Minch [42]. The prevalence from Gondar (5.5%) [43] and Jimma (4.4%) [44] is in line with this study. The probable reason for the variations in the prevalence of *Salmonella* between studies could be due to differences in sample size, sampling techniques, laboratory procedures, study areas, seasons, and hygienic conditions employed [45].

Although there is a difference in prevalence, the trend of an increase in *Salmonella* isolation in the current study from abattoirs (4.24%) and butchers (8.47%) was similar to the results from Addis Ababa abattoir (26.3%) and butchers (32.4%) [38]. The significant variation in the prevalence between the abattoirs and the butcher shops might be due to the risk of direct carcass cross-contamination during loading and transportation of the carcass using a single car and unhygienic handling of meat at butcher shops (personal observation). Abattoir workers carry the meat on their back or chest, hold, and support it using their two hands. Therefore, the unhygienic handling practice of meat and direct contact between contaminated clothes of loader and carcass might be the cause for the higher prevalence of *Salmonella* from the butcher shops. Furthermore, all the butcher shops are located along the major roadsides, and meat displayed for sale was not covered thus exposing the meat to contamination by flies and dust. On the contrary, the relatively lower prevalence of *Salmonella* in meat samples from restaurants (“Kitfo”) might be because meat for “Kitfo” preparation was taken from the softer part following the removal of some layer of meat which might have been exposed to contamination.

*S. typhimurium*, the dominant serotypes isolated in the current study, is one of the common causes of nontyphoidal salmonellosis in people [46]. The three top-ranked *Salmonella* serotypes commonly identified from cattle slaughtered in Addis Ababa are *S. mishmarhaemek*, *S. typhimurium*, and *S*. *enteritidis* [47]. In a different study carried out in northern Ethiopia from slaughtered cattle, *S. typhimurium* and *S. newport* were the two dominant serotypes isolated [41]. The other serotype detected in this study was *S. enterica* serotype 1,4,5,12: i:-, a monophasic variant of *Salmonella typhimurium*, which has also been isolated previously from slaughtered cattle carcass swabs, cecal contents, and slaughterhouse environment from southern Ethiopia [33]. This serotype is emerging in recent years and has become one of the most common serotypes isolated from humans and swine enteric salmonellosis [48]. Even though there is limited diversity in the serotypes in the current study, it is believed that this study adds to the global knowledge on the occurrence, risk factors, and antimicrobial resistance particularly concerning the emerging *Salmonella enterica* serotype 1,4,5,12: i:-.

Antimicrobial resistance is an emerging global problem in human and veterinary medicine in both developed and developing nations. The important factor for the production of bacterial resistance is the expanded utilization of antimicrobial agents in food animal production and humans [49]. In the present study, all *Salmonella* isolates were highly susceptible to cotrimoxazole, chloramphenicol, norfloxacin, and nitrofurantoin. This is in line with the findings from food handlers in Arba Minch University student's lunchroom [50], food handlers of Gondar University student lunchroom [19], dairy farms in Modjo [51], and from milk samples from Addis Ababa [52]. In this study, all the *Salmonella* isolates were susceptible to cotrimoxazole unlike the other reports from different parts of Ethiopia where resistance to cotrimoxazole ranging from 34.5 to 100% has been reported [42, 53, 54].

The current study revealed that *Salmonella* isolates were resistant to azithromycin (40%), amoxicillin (30%), ceftriaxone (20%), ceftazidime (20%), nalidixic acid (25%), and tetracycline (15%). In line with this, the ﬁrst failure of azithromycin therapy was reported in 2010 in a patient with invasive *Salmonella* infection [55]. The resistance rate of the isolates to amoxicillin in this study was less than the 100% resistance reported in the Nekemte Referral Hospital [56], and the 69.2% reported in Gondar University [19]. Similarly, much lower resistance to ampicillin (5%) and tetracycline (15%) was recorded in the current study as compared to previous investigations where resistances ranging from 82.3–100% for ampicillin [32, 37, 57] and 47.4% for tetracycline [37] have been reported. The 22% resistance rate to nalidixic acid in this study was similar to that of Lamboro et al. [37].

In the current study, both serotypes identified were resistant to different antimicrobials. Of the 20 isolates, 6 (30%) were resistant to three or more classes of antimicrobial drugs, while 9 isolates (45%) were resistant to at least two classes of drugs. The percentage of the MDR isolates in the current study is less than the previous reports, which ranges from 75.5% to 84.6% MDR [19, 32, 53]. Since most of these drugs are also commonly used in human medicine in Ethiopia, this resistance to antimicrobials is highly significant. For instance, ciprofloxacin and ceftriaxone are used for the treatment of bacillary dysentery; chloramphenicol, trimethoprim/sulfamethoxazole, and ciprofloxacin are used against gastroenteritis; the latter two drugs are also used to treat cholera; amoxicillin, gentamycin, and ceftriaxone are used to treat pneumonia; trimethoprim/sulfamethoxazole and amoxicillin are used against sinusitis [58]. Moreover, it has been observed that prescriptions are usually made without prior isolation and performing drug susceptibility tests for infectious agents in the study areas. Furthermore, the possible lateral transmission of resistance traits and other virulence factors or plasmids from *Salmonella* to different microorganisms, inside the human gut, may exist. Resistance traits in *Salmonella* can be hereditarily decided and may include chromosomal mutations or plasmid-mediated and might be exchanged with other *Enterobacteriaceae* species [59]. There is a lack of advocacy and monitoring of antimicrobial drug utilization at all levels in Ethiopia. Additionally, underdose or prophylactic usage of the drugs in food animals may produce antimicrobial resistance genes in *Salmonella* as well as other potential human and animal pathogens.

The high antimicrobial resistance rates observed in this study for some drugs might have been due to the natural process or could be because of the uncontrolled accessibility of the antimicrobial agents in drug vendors, which prompts abuse and greater selection pressure for resistant strains. It could also be due to the deficiency or nonappearance of antimicrobial resistance observation programs [60, 61]. The presence of antimicrobial resistance adversely affects human health by causing severe illness that is more difficult and expensive to treat. This is because resistant infections are more severe and those patients are more likely to be hospitalized, and treatment in such cases is less effective [49].

The study noted that 79.6% of the respondent had information about food safety from nonformal ways such as mass media, friends, and parents. The previous study indicated that proper training, education, and monitoring of the workers could address the limitation of the implementation of personal hygienic practices [62]. Despite most respondents in the present study (92.6%) did not receive any formal training regarding hygiene and sanitation of meat handling neither prior nor after employment, 66.7% of the abattoirs, 73.7% of butchers, and 50.8% of restaurants workers knew contaminated meat could cause bacterial diseases (Table 7). A study conducted in Malaysia reported that 73.4% of food handlers had acceptable knowledge of food-borne pathogens [63]. In this study, the knowledge of the respondent about food hygiene and safety as well as the food-borne diseases causing microorganisms is 62.8%, which is less than the report mentioned in Malaysia. Since meat handlers can serve as vehicles for cross-contamination and the spread of food-borne pathogens, they need to know the importance of proper meat handling, hand washing, and other important hygienic procedures [63]. With this regard, it has been well documented that training enhances food handlers' awareness of food-borne diseases and could enable them to better understand and fulfill their responsibilities and exercise skills [64]. This study also showed that the odds of good food hygiene practices were higher among food handlers who had formal education as compared to those who had not. Accordingly, the odds ratio of isolating *Salmonella* was 7.2 times (AOR: 7.2, 95% CI 1.2–51.2) higher in butchers and restaurants where the workers did not attend any formal education compared to those who attended secondary school and above. In line with this finding, a study conducted by Asrat et al. [65] in Ethiopia also revealed the importance of education for food handlers to ensure food safety.

Wiping cloth was used for cleaning purposes particularly at butcher shops and restaurants in the current study, and the intention was good. However, it was reused the whole day and can accumulate microorganisms that can be transferred to the retailer operators' hands, to utensil surfaces, and finally to meat. Clothes have also been reported to be ineffective in removing microorganisms, thereby increasing the chance of cross-contamination [66].

There was improper disposal of leftover dirty materials (gastrointestinal content, horn, shank, and bones) collected from daily slaughtered animals at the abattoirs. The floor of the abattoir ought to be hard concrete and impenetrable, to minimize dirt and permit seepage and simplicity of cleaning. Likewise, a rooftop is critical to shield the carcass from the weather and to diminish the temperature in the abattoir [67]. In the present study, the floor of the abattoirs was made of concrete and impervious material but has no ceiling. Even though washing of the floor takes place every day at the end of the slaughtering process, the slaughter wall is not cleaned and washed at the end of the working day. Although different factors were there for cross-contamination of the meat, the training of the workers, the educational background of the meat handlers, and the food safety and hygiene knowledge of the meat handlers play a vital role.

The prevalence of *Salmonella* from raw meat in this study, though low, could give a cautionary signal for the conceivable event of food-borne diseases capable of causing outbreaks and pose a public health risk through the exported meat and meat products to the international market. The poor sanitation of butchers/restaurants, the poor hygiene of the slicing materials, the cutting boards, and the food handler's surfaces might have contributed to the reported prevalence of *Salmonella* in the current study. The absence of washing basins and first aid kits in butchers and restaurants might be attributed to the failure of local authorities to enforce existing food-related laws, lack of resources, and information about hygienic practices as the vast majority of the laborers had no formal training in sanitation. Poor individual cleanliness is one of the most significant sources of contamination for foods. Therefore, control measures along the meat processing chain, namely, abattoirs, meat processing plants, distributors, and consumers, should be undertaken to minimize the risk of cross-contamination and food-borne infections caused by *Salmonella* spp. [68].

## 5. Conclusion

The current study showed the highest prevalence of *Salmonella* isolates from butcher shops. The dominant serotype was *S. typhimurium*. The high rate of MDR *Salmonella* suggests a potential health risk to consumers from the consumption of raw meat. Education, knowledge of food safety, hygiene, and training on food safety and hygiene of workers are predictors of *Salmonella* isolation. The poor meat handling and poor personal hygienic practices of workers in the retail shops and restaurants may pose a risk of food-borne disease. Therefore, the use of soft, absorbent disposable paper towels for drying hands instead of cloth, continuous food safety education, and training for meat handlers are recommended to enhance good safety practices. There is a need to emphasize discouraging raw meat consumption and indiscriminate use of antimicrobial drugs. Besides, regular antimicrobial susceptibility surveillance is essential.

## Figures and Tables

**Table 1:** Prevalence of *Salmonella* in abattoirs, butchers, and restaurants of Ambo and Holeta towns.

Sources of meat	Ambo town		Holeta town		Total	
	No. examined	*Salmonella* positive (%)	No. examined	*Salmonella* positive (%)	No. examined	Prevalence (%)
Abattoirs	89	5 (5.6)	29	0 (0)	118	4.2
Butchers	89	10 (11.2)	29	0 (0)	118	8.5
Restaurants	89	5 (5.6)	29	0 (0)	118	4.2
Total	267	20 (7.5)	87	0 (0)	354	5.7

**Table 2:** *Salmonella* serotypes distribution and site of isolation.

Serotypes	Site of isolation			
	Abattoirs	Butchers	Restaurants	Total
*S. typhimurium*	3	3	5	11
I:4,5,12:i:-	2	7	0	9
Total	5	10	5	20

**Table 3:** Antimicrobial susceptibility test of *Salmonella* isolates from meat samples of Ambo and Holeta towns, central Ethiopia.

Antimicrobial class	Antimicrobial discs and concentration	*Salmonella* isolates (*n* = 20)		
		No. of susceptible isolates (%)	No. of. intermediate isolates (%)	No. of. resistant isolates (%)
Aminoglycosides	Amikacin (30 *μ*g)	15 (75.0)	5 (25.0)	0 (0.0)
	Gentamicin (10 *μ*g)	16 (80.0)	2 (10.0)	2 (10.0)
Cephems	Cefotaxime (30 *μ*g)	17 (85.0)	1 (5.0)	2 (10.0)
	Ceftazidime (30 *μ*g)	16 (80.0)	0 (0.0)	4 (20.0)
	Ceftriaxone (5 *μ*g)	15 (75.0)	1 (5.0)	4 (20.0)
Macrolide	Azithromycin (30 *μ*g)	3 (15.0)	9 (45.0)	8 (40.0)
Nitrofurans	Nitrofurantoin (300 *μ*g)	16 (80.0)	1 (5.0)	3 (15.0)
Phenicols	Chloramphenicol (30 *μ*g)	19 (95.0)	0 (0.0)	1 (5.0)
Quinolones	Ciprofloxacin (5 *μ*g)	12 (60.0)	8 (40.0)	0 (0.0)
	Nalidixic acid (30 *μ*g)	15 (75.0)	0 (0.0)	5 (25.0)
	Norfloxacin (10 *μ*g)	19 (95.0)	1 (5.0)	0 (0.0)
Sulphonamides	Cotrimoxazole (25 *μ*g)	20 (100.0)	0 (0.0)	0 (0.0)
Tetracycline	Tetracycline (30 *μ*g)	17 (85.0)	0 (0.0)	3 (15.0)
*β*-Lactams	Ampicillin (10 *μ*g)	18 (90.0)	1 (5.0)	1 (5.0)
	Amoxicillin (25 *μ*g)	5 (25.0)	9 (45.0)	6 (30.0)

**Table 4:** Multidrug resistance patterns in *Salmonella* isolated from meat samples in Ambo and Holeta towns.

Number	Antimicrobial resistance pattern	No. of resistant isolates (%)
Two	AMX, AZM	3 (9.7)
	AZM, TET	3 (9.7)
	NA,CTR	3 (9.7)
	NA, CAZ	3 (9.7)
	AMX, TET	2 (6.5)
	AMX, NA	2 (6.5)
	AMX, CTR	2 (6.5)
	AZM, GEN	2 (6.5)
	AMX, AZM, NIT	2 (6.5)
	AMX, AZM, TET	2 (6.5)

Three	AZM, NIT, TET	2 (6.5)
	AMX, CXT, NA	1 (3.2)
	AZM, CXT, NA	1 (3.2)

Four	AMX, AZM, NIT, TET	1 (3.2)
	AZM, CHL, NIT, TET	1 (3.2)

Five	AMX, AZM, GEN, NA, TET	1 (3.2)

Total		31 (100%)

AZM: azithromycin; AMX: amoxicillin; CTR: ceftriaxone; TET: tetracycline; NA: nalidixic acid; CXT: cefotaxime; CHL: chloramphenicol; NIT: nitrofurantoin; GEN: gentamycin.

**Table 5:** Sociodemographic characteristics of abattoir, butcher, and restaurant workers in Ambo and Holeta towns.

Variables	Categories	Abattoir workers		Butcher workers		Restaurant workers	
		Freq.	%	Freq.	%	Freq.	%
Age in years	Less than 20	7	21.2	14	11.9	34	28.8
	21–29	22	66.7	61	51.7	60	50.9
	30–38	2	6.1	31	26.3	24	20.3
	≥39	2	6.1	12	10.2	0	0

Sex	Male	33	100	118	100	6	5.1
	Female	0	0	0	0	112	94.9

Religion	Orthodox	21	63.	111	94.1	75	63.6
	Protestant	10	30.30	7	5.9	40	33.9
	Others	2	6.1	0	0	3	2.5

Marital status	Single	15	45.5	45	38.1	61	51.7
	Married	18	54.6	73	61.9	54	45.8
	Divorced	0	0	0	0	3	2.5

Residence	Urban	26	78.8	77	65.3	53	44.9
	Rural	7	21.2	41	34.8	65	55.1

Education	Illiterate	3	9.1	9	7.6	18	15.3
	Primary (1–8)	17	51.5	67	56.8	80	67.8
	Secondary (9–12)	6	18.2	34	28.8	20	17.0
	Tertiary	7	21.2	8	6.8	0	0

Freq. = frequency.

**Table 6:** Knowledge and practice on food hygiene and food safety.

Variables	Categories	Abattoir workers		Butcher workers		Restaurants workers	
		Freq.	%	Freq.	%	Freq.	%
Have food hygiene and safety information	Yes	31	93.9	85	72.0	98	83.1
	No	2	6.1	33	28.0	20	16.9

Has training on food safety and hygiene	Yes	5	15.2	27	22.9	9	7.6
	No	28	84.9	91	77.1	110	92.4

Knows food safety and hygiene	Yes	22	66.7	53	44.9	78	66.1
	No	11	33.3	65	55.1	40	33.9

Practice food safety and hygiene	Yes	15	45.5	50	42.4	81	68.6
	No	18	54.6	68	57.6	37	31.3

**Table 7:** Univariable logistic regression analysis of factors associated with meat contamination with *Salmonella* in butcher and restaurants of Ambo and Holeta towns.

Variables	Categories	Tested	Pos.	%	OR	95% CI	*P* value
Source	Restaurants	118	5	4.2	1.0		
	Butchers	118	10	8.5	2.0	0.7–6.3	0.110

Sex	Female	124	5	4.5	1.0		
	Male	112	10	8.1	1.9	0.6–5.7	0.264

Age in years	≤24	89	2	2.1	1.0		
	25–30	96	9	9.4	3.7	0.7–21.0	0.139
	≥31	51	4	7.8	4.5	0.9–21.4	0.059

Marital status	Single	106	6	5.7	1.0		
	Married and divorced	130	9	6.9	1.2	0.4–3.3	0.693

Residence	Rural	106	6	5.7	1.0		
	Urban	130	9	6.9	1.2	0.4–3.3	0.693

Religion	Orthodox	186	8	4.3	1.0		
	Protestant	50	7	14	3.6	1.3–10.5	0.018

Education	Secondary and above	27	5	18.5	1.0		
	Primary	147	8	5.4	1.7	0.4–8.4	0.498
	Illiterate	62	2	3.2	6.8	1.2–37.7	0.028

Experience in years	<1	51	2	3.9	1.0		
	1–3	133	9	6.8	1.8	0.4–8.5	0.472
	≥4	52	4	7.7	2.0	0.4–11.7	0.422

Has information about food safety and hygiene	No	183	13	7.1	1.0		
	Yes	53	2	3.8	1.9	0.4–8.9	0.390

As training on food safety and hygiene	Yes	35	7	20	1.0		
	No	201	8	4.0	6.0	2.0–17.9	0.001

Time spend on the work per day	8–12 hrs	122	2	1.6	1.0		
	≥13 hrs	114	13	11.4	7.7	1.7–35.0	0.008

Knows food safety and hygiene	Yes	131	2	1.5	1.0		
	No	105	13	12.4	9.1	2.0–41.4	0.004

Practice food safety and hygiene	Yes	131	7	5.3	1.0		
	No	105	8	7.6	1.5	0.5–4.2	0.479

Sanitation of butcher/restaurants	Fair	26	5	19.2	1.0		
	Good	127	5	3.9	1.6	0.4–5.6	0.491
	Poor	83	5	4.8	5.8	1.6–21.8	0.009

Hygiene of slicing material	Fair	14	4	28.6	1.0		
	Good	146	7	4.8	1.1	0.3–3.9	0.879
	Poor	76	4	5.3	7.94	2.0–31.8	0.003

Hygiene of cutting board	Good	18	3	16.7	1.0		
	Fair	127	7	5.5	1.0	0.3–3.3	0.996
	Poor	91	5	5.5	4.4	0.7–15.9	0.114

Hygiene of food handlers	Good	24	6	25.0	1.0		
	Fair	114	6	5.3	1.8	0.4–7.2	0.433
	Poor	98	3	3.1	10	2.5–46.1	0.002

Refrigerator using	Yes	222	14	6.4	1.0		
	No	14	1	7.1	1.1	0.1–9.4	0.901

Presence of insect	No	187	12	6.4	1.0		
	Yes	49	3	6.1	1.1	0.3–3.9	0.940

Presence of rodents	Yes	53	2	1.9	1.0		
	No	183	13	7.1	1.9	0.4–8.9	0.390

**Table 8:** Multivariable logistic regression analysis of factors associated with the contamination of meat with *Salmonella* in Ambo and Holeta towns, central Ethiopia.

Variables	Categories	AOR	95% CI	*P* value
Age in years	≤24	1.0		
	25–30	2.8	0.4–21.1	0.315
	≥31	6.5	1.1–39.3	**0.040**

Education	Secondary and above	1.0		
	Primary	1.68	0.3–10.5	0.578
	Illiterate	8.0	1.0–64.3	0.**049**

Has training on food safety and hygiene	Yes	1.0		
	No	5.7	1.6–20.8	**0.008**

Knows food safety and hygiene	Yes	1.0		
	No	10.52	1.9–57.2	0.**006**

Sanitation of butcher/restaurants	Fair	1.0		
	Good	3.69	0.9–15.8	0.078
	Poor	4.16	0.8–22.3	0.094

AOR = adjusted odds ratio; CI = confidence interval. The values in bold are statistically significant.

**Table 9:** Best-fitting model for predictors of *Salmonella* isolation in butchers and restaurants of Ambo and Holeta towns, central Ethiopia.

Variables	Categories	AOR	95% CI	*P* value
Education	Secondary and above	1.0		
	Primary	1.6	0.3–8.3	0.582
	Illiterate	7.8	1.2–51.2	0.**032**

Has training on food safety and hygiene	Yes	1.0		
	No	6.3	1.9–21.2	**0.003**

Knows food safety and hygiene	Yes	1.0		
	No	7.6	1.6–35.9	0.**010**

The values in bold are statistically significant.

## Data Availability

The datasets used and/or analyzed during the current research are available from the corresponding author on reasonable request.
